# The Effect of Intermittent Pneumatic Compression on Hemodynamics and Regional Cerebral Oxygen Saturation in Laparoscopic Bariatric Surgery with Mild Hypercapnia in the Steep Reverse Trendelenburg Position

**DOI:** 10.3390/jpm14040405

**Published:** 2024-04-11

**Authors:** Youn Yi Jo, Seong Min Kim, Chun Gon Park, Ji Woong Kim, Hyun Jeong Kwak

**Affiliations:** 1 Department of Anesthesiology and Pain Medicine, College of Medicine, Gil Hospital, Gachon University, Incheon 21565, Republic of Korea; endless37@gilhospital.com (Y.Y.J.); chungony@gilhospital.com (C.G.P.); 19709@gilhospital.com (J.W.K.); 2 Department of Surgery, College of Medicine, Gil Hospital, Gachon University, Incheon 21565, Republic of Korea; seongmin_kim@gilhospital.com

**Keywords:** obesity, intermittent pneumatic compression, hemodynamic, cerebral desaturation

## Abstract

Obesity negatively affects hemodynamics and cerebral physiology. We investigated the effect of the utilization of an intermittent pneumatic compression (IPC) device on hemodynamics and cerebral physiology in patients undergoing laparoscopic bariatric surgery under general anesthesia with lung-protective ventilation. Sixty-four patients (body mass index > 30 kg/m^2^) were randomly assigned to groups that received an IPC device (IPC group, *n* = 32) and did not (control group, *n* = 32). The mean arterial pressure (MAP), heart rate (HR), need for vasopressors, cerebral oxygen saturation (rSO_2_), and cerebral desaturation events were recorded. The incidence of intraoperative hypotension was not significantly different between groups (*p* = 0.153). Changes in MAP and HR over time were similar between groups (*p* = 0.196 and *p* = 0.705, respectively). The incidence of intraoperative cerebral desaturation was not significantly different between groups (*p* = 0.488). Changes in rSO_2_ over time were similar between the two groups (*p* = 0.190) during pneumoperitoneum. Applying IPC to patients with obesity in the steep reverse Trendelenburg position may not improve hemodynamic parameters, vasopressor requirements, or rSO_2_ values during pneumoperitoneum under lung-protective ventilation. During laparoscopic bariatric surgery, IPC alone has limitations in improving hemodynamics and cerebral physiology.

## 1. Introduction

Obesity negatively affects patient hemodynamics and cerebral physiology during anesthesia and surgery. Morbid obesity increases the levels of vasoactive adipokines, leading to a reduction in systemic vascular resistance due to vasodilation [1]. A previous study reported that the mean daily brain tissue oxygen tension in patients with severe brain injury was significantly lower in patients with obesity than in those without (25.8 mmHg vs. 31.8 mmHg) [2]. In addition, in a multivariate regression analysis, the body mass index (BMI) was significantly correlated with the deterioration of brain tissue oxygen tension. This is presumed to be due to obesity-related pulmonary dysfunction or the impairment of compensatory mechanisms caused by changes in chronic metabolic or inflammatory responses [2]. The reverse Trendelenburg position or sitting position, in which a patient’s head is positioned higher than their feet, can further exacerbate the negative cerebral effects by reducing blood pressure and cerebral blood flow [3]. A previous clinical study showed that 80% of patients under general anesthesia experienced a cerebral desaturation event, which is caused by a decrease in cerebral perfusion pressure (CPP), when adopting the head-up position from supine [4]. Laparoscopic surgery using intraabdominal carbon dioxide (CO_2_) insufflation or hypercapnia for lung-protective ventilation may alleviate cerebral desaturation in the reverse Trendelenburg position [5]. The effects of hypercapnia on the brain are complex. Hypercapnia can increase cerebral blood volume by causing cerebral vasodilation and decrease CPP by increasing intracranial pressure, which can also alter cerebral oxygen saturation [6].

An intermittent pneumatic compression (IPC) device is a device worn on the lower extremities to prevent deep venous thrombosis (DVT). Since patients with obesity frequently have venous impairment, they are vulnerable to venous thromboembolism, and mechanical prophylaxis such as lower-extremity pneumatic compression is often recommended [7]. Aside from their effectiveness in preventing venous thromboembolism, IPC devices have been studied to improve cerebral oxygen saturation by increasing venous return from the lower extremities and contribute to hemodynamic stability [8,9]. In a study conducted on 70 patients under general anesthesia, pneumatic compression of the lower extremities significantly reduced the incidence of hypotension and the median cumulative hypotension time compared with no compression [10].

Previous studies have reported the effects of the head-up position or IPC on hemodynamics and cerebral physiology under general anesthesia [3,4,8,9,10]. However, to date, reports on the effect of lower-extremity IPC on hemodynamics and cerebral oxygen saturation in the reverse Trendelenburg position in patients with obesity undergoing laparoscopic surgery are lacking. It was hypothesized that utilizing the IPC device in patients with obesity undergoing laparoscopic surgery would not only reduce the occurrence of hypotension due to the steep reverse Trendelenburg position but also improve cerebral oxygen saturation. Therefore, we investigated the effect of utilizing an IPC device on hemodynamics and cerebral physiology in patients undergoing laparoscopic bariatric surgery under general anesthesia with lung-protective ventilation. We compared those who received IPC with those who did not.

## 2. Materials and Methods

This prospective randomized trial, registered at cris.nih.go.kr (KCT0007095), was approved by the Institutional Review Board (IRB) of Gachon University Gil Hospital (approval no: GBIRB2022-023). Before starting the trial, all the participants provided informed consent. A total of 64 patients with obesity aged 19–65 years with an American Society of Anesthesiologists (ASA) physical status of 2 (body mass index; BMI > 30 kg/m^2^) scheduled for laparoscopic sleeve gastrectomy were included. Patients with a history of autonomic nervous system dysfunction; cerebrovascular disease; uncontrolled cardiovascular disease, including hypertension; uncontrolled endocrine disorders, including diabetes mellitus; peripheral vascular disease; and uncontrolled respiratory disease were excluded. The participants were randomly assigned to a group that received the IPC device (IPC group, *n* = 32) and a group that did not (control group, *n* = 32) using a randomization process conducted in Excel 2013 (Microsoft Office, Redmond, WA, USA). The IPC device (Kendal SCDTM 700 series, Covidien, MA, USA) was used in the IPC group after anesthesia induction. The postoperative outcome assistant and patients were blinded to the group assignment; only the anesthesiologists conducting the study were aware of the group assignment.

No analgesics or sedatives were administered as premedications. In the operating room, a pulse oximeter, an electrocardiogram, and the non-invasive blood pressure were monitored. A near-infrared spectroscopy sensor was used to measure regional cerebral oxygen saturation (rSO_2_), while a CONOX sensor (Quantium Medical, Spain/Fresenius Kabi, Germany) was used to measure the depth of anesthesia; these were attached to patients’ foreheads prior to anesthesia induction. For anesthesia induction, 1 mg/kg lidocaine, 1.5–2.0 mg/kg propofol, 0.5–1.0 µg/kg remifentanil, and 0.8 mg/kg rocuronium were administered. Sevoflurane at a concentration of 1.5–2.5 vol% and remifentanil at a rate of 0.05–0.2 µg/kg/min were administered for anesthesia maintenance. Sevoflurane was adjusted to maintain the quantitative consciousness index (qCON) in CONOX at 30–60, and remifentanil was adjusted to maintain the quantitative nociception index (qNOX) in CONOX at 30–60. The pressure-controlled ventilation volume guarantee mode with an inspired oxygen fraction of 0.6 was applied. For mechanical ventilation, a tidal volume of 6 mL/kg of ideal body weight, an inspiratory to expiratory (I:E) ratio of 1:1, and an external positive end-expiratory pressure of 5 cmH_2_O were used. The ideal body weight was calculated using the following formula: 0.919 × (height in cm − 152.4) + 50 for men or + 45.5 for women. To maintain an end-tidal carbon dioxide tension (ETCO_2_) of 40 ± 3 mmHg, the respiratory rate was adjusted. A radial arterial line was inserted after the induction of anesthesia. For laparoscopy, pneumoperitoneum with abdominal insufflation of CO_2_ gas at 16–18 mmHg and a steep reverse Trendelenburg position at 60° were adopted.

During anesthesia, the mean arterial pressure (MAP), heart rate (HR), use of a vasopressor, qCOX, qNOX, peak airway pressure, tidal volume, respiratory rate, ETCO_2_, peripheral capillary oxygen saturation (SpO_2_), and rSO_2_ were recorded at the following time points: before (baseline) and at 5 min after anesthesia induction (IND5) in supine, and at 1, 3, 5, 10, and 20 min after pneumoperitoneum (PP1, PP3, PP5, PP10, and PP20, respectively) in the 60° reverse Trendelenburg position. Arterial blood gas analysis was performed at IND5 and PP20. Hypotension was treated with 5 mg of intravenous ephedrine or 100 µg of phenylephrine as appropriate when MAP dropped below 80% of the baseline value or when systolic blood pressure dropped below 90 mmHg. Cerebral desaturation was defined as the mean of the right and left rSO_2_ dropping below 80% of the baseline value or dropping below the absolute value of 50%.

The primary outcome was the incidence of hypotension after reverse Trendelenburg positioning. Secondary outcomes were changes in hemodynamics and rSO_2_ values during pneumoperitoneum in the steep reverse Trendelenburg position. The sample size was calculated based on a previous study [11], which showed that the use of an IPC device after sitting reduced the incidence of hypotension from 64% to 28%. Each group required 29 patients, with a power of 80% and an error of 0.05. Thus, assuming a 10% dropout rate, 64 patients were recruited for this study.

SPSS (version 22.0; SPSS Inc., Chicago, IL, USA) was used for statistical analyses. To assess the normality of the distribution of continuous variables, the Kolmogorov–Smirnov test was performed. Comparison of continuous variables was conducted using the independent t-test or Mann–Whitney U test, as appropriate, and presented as the mean ± standard deviation. Comparison of categorical data was performed using the x^2^-test or Fisher’s exact test, as appropriate, and presented as the number (%). Changes in the variables over time were compared using repeated-measures analysis of variance (ANOVA). *p* values < 0.05 were considered statistically significant.

## 3. Results

### 3.1. Participants

**Figure 1:**
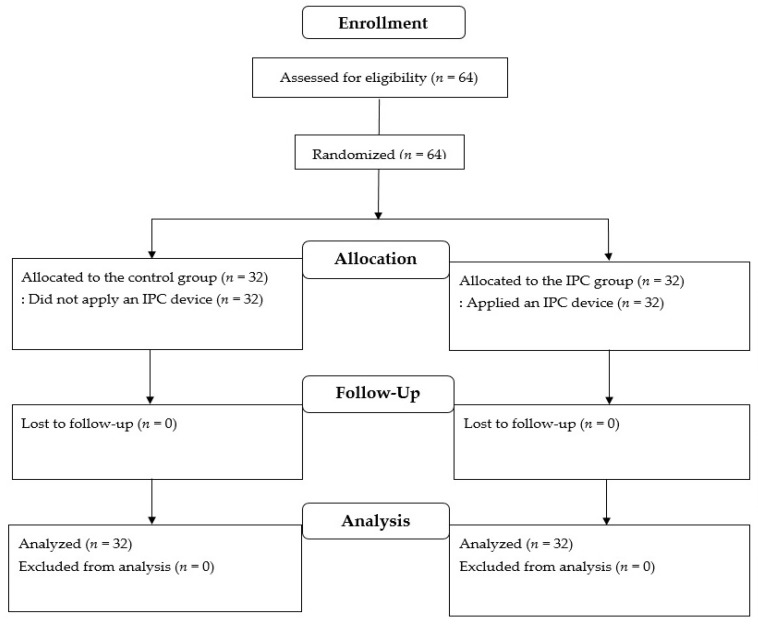
Flow diagram depicting patient allocation.

All 64 participants that were enrolled in this study were included in the statistical analyses; there were no dropouts (Figure 1). Patient characteristics, including BMI, were similar between the two groups. Perioperative data, including operative time and hospital stay, were similar between the two groups (Table 1). Numbered lists can be added as follows:

### 3.2. Intraoperative Hemodynamic Data

**Figure 2:**
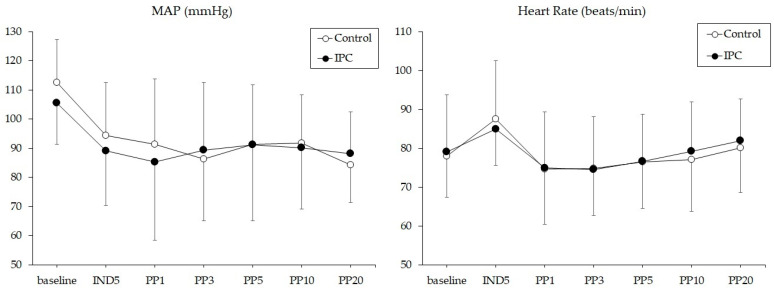
Changes in mean arterial pressure (MAP) and heart rate (HR) during surgery. Control, not applying intermittent pneumatic compression device (IPC); IPC, applying IPC.

The incidence of intraoperative hypotension requiring vasopressors such as ephedrine or phenylephrine was not significantly different between the control and IPC groups (15 patients (47%) vs. 10 patients (31%), *p* = 0.153). The changes in MAP and HR are illustrated in Figure 2. Changes in MAP and HR over time were similar between the control and IPC groups (*p* = 0.196 and *p* = 0.705, respectively). There were no intergroup differences in the MAP or HR at any study time point.

### 3.3. Chanages in ETCO_2_ and rSO_2_

**Figure 3:**
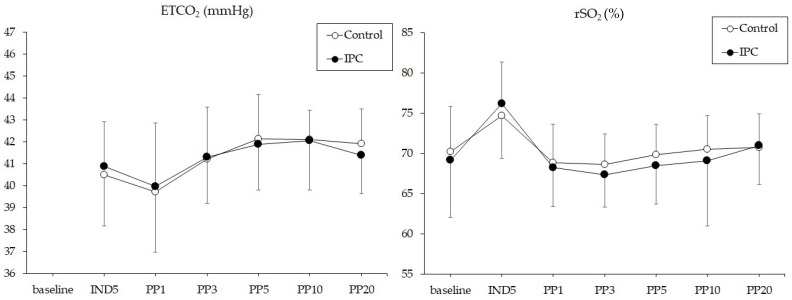
Changes in end-tidal carbon dioxide tension (ETCO_2_) and regional cerebral oxygen saturation (rSO_2_) during surgery. Control, not applying intermittent pneumatic compression device (IPC); IPC, applying IPC.

The incidence of intraoperative cerebral desaturation was not significantly different between the control and IPC groups (one patient (3%) vs. two patients (6%), *p* = 0.488). None of the patients experienced an absolute rSO_2_ value of <50% or postoperative cognitive dysfunction. The changes in rSO_2_ and ETCO_2_ are illustrated in Figure 3. Changes in rSO_2_ and ETCO_2_ over time were similar between the control and IPC groups (*p* = 0.190 and *p* = 0.756, respectively). There were no intergroup differences in rSO_2_ or ETCO_2_ at any time point. In both the control and IPC groups, rSO_2_ increased significantly at IND5 (*p* = 0.003 and *p* < 0.001, respectively) compared to the baseline values, whereas it decreased significantly at PP1, 3, 5, 10, and 20 (all *p* < 0.05) compared to IND5.

The changes in respiratory parameters (not illustrated) over time, including peak airway pressure, tidal volume, respiratory rate, SpO_2_ and dynamic compliance, were similar between the two groups. No intergroup differences were observed in any of the respiratory parameters at any time point. 

## 4. Discussion

This study demonstrated that the application of IPC in patients with obesity in the steep reverse Trendelenburg position did not alleviate the occurrence of intraoperative hypotension or decrease rSO_2_ during CO_2_ pneumoperitoneum under lung-protective ventilation.

Patients with obesity have increased intra-abdominal pressure due to excessive visceral fat, which leads to the requirement of higher intraluminal pressure to maintain proper transmural distending pressure [12]. According to a previous clinical study, visceral adipose tissue is 34% more abundant in women with heart failure and preserved left ventricular ejection fraction (HFpEF) than in those without HFpEF [12]. Approximately 80% of the patients are obese [13], and obesity-related HFpEF can manifest as various hemodynamic changes. Excessive body fat disrupts the autonomic nervous system and can cause abnormalities in arterial baroreflex sensitivity [14]. When the negative effects of obesity-related increased abdominal pressure are already evident, an additional increase in abdominal pressure due to intra-abdominal CO_2_ insufflation during laparoscopic surgery may further worsen the cardiovascular response. In this study, a decreasing trend in MAP and a significant decrease in HR were observed immediately after pneumoperitoneum and positional changes and after pneumoperitoneum, respectively, in both of the groups.

IPC prevents DVT by preventing venous stasis and inhibiting hypercoagulability by increasing venous blood flow velocity and increasing fibrinolytic activity, respectively [15]. Changes in blood distribution induced by general anesthesia or postural changes can cause hemodynamic instability. External compression of the lower extremities reduces peripheral pooling of blood, which increases blood flow to the central compartment, preserving preload and contributing to hemodynamic stability [8,9]. The total blood volume is divided into unstressed blood volume (UBV) and stressed blood volume (SBV). UBV refers to the volume that fills the blood vessel to the point where intravascular pressure and wall tension occur, and SBV refers to the excess volume of UBV that increases wall tension and changes the pressure [16]. The fact that a change in SBV leads to an increase in pressure depends on the compliance and capacitance of the central vein and its ability to discharge blood from the right heart to the pulmonary lungs [16]. However, an increase in SBV during rest and exercise was greater in patients with obesity than in those without, indicating reduced central venous compliance and impaired right ventricle-pulmonary artery (RV-PA) coupling [16,17]. Reduced central venous compliance and impaired RV-PA coupling in patients with obesity may have offset the effect of IPC in enhancing venous return in this study. In a previous study of patients undergoing laparoscopy, IPC increased cardiac output and stroke volume, which were reduced by pneumoperitoneum and the head-up position by 27% and 16%, respectively [18]. However, our study targeted patients with obesity, and their basal abdominal pressure was higher than that of patients without obesity [12], which seems to be one of the factors that offsets the positive hemodynamic effects of IPC.

A clinical study demonstrated that IPC was effective in recovering from rSO_2_ deterioration experienced during laparoscopic cholecystectomy [8]. They explained that the increase in intra-abdominal pressure during pneumoperitoneum reduces stroke volume and cardiac output and increases CVP and that IPC alleviates decreased rSO_2_ by improving venous return [8]. This study confirmed that the application of IPC significantly reduced HR compared to no application, demonstrating the effect of improving venous return [8]. However, in our study, there was no significant correlation between IPC application and changes in HR, and there was no difference in rSO_2_ between the two groups. In patients with obesity, changes in CVP are limited due to limited venous compliance [16], and considering that obesity and CVP are independent factors that compromise brain tissue oxygen tension [2], this might be a consequence of similar rSO_2_, regardless of IPC application in this study.

Mild hypercapnia was induced as a lung-protective ventilator strategy in both groups. Previous clinical studies regarding laparoscopic surgery showed that mild hypercapnia did not worsen the increased rSO_2_ caused by ICP but improved the value of rSO_2_ [19,20]. Although this was not a comparative study observing the effects of mild hypercapnia, we expected that mild hypercapnia for lung protective ventilation might attenuate the deleterious effects on cerebral physiology caused by obesity, increased intra-abdominal pressure, and steep reverse Trendelenburg positioning. In this study, when factors that affect rSO_2_, such as respiratory parameters, arterial gas tension, and hematocrit, were similar, IPC alone appeared to have limitations in inducing changes in rSO_2_ by changing hemodynamic parameters.

Laparoscopic surgery has become the mainstream of abdominal surgery because it is less invasive, leads to a faster recovery, and shows improved morbidity and mortality compared to open surgery [21]. The usefulness of laparoscopic surgery has been proven not only in elective surgery, such as cancer surgery, but also in emergency surgery, including trauma [22]. Since laparoscopic surgery is used in high-risk patients such as elderly patients, patients with underlying diseases, patients with obesity, and in emergency situations such as trauma, it is more important to attempt to ensure the stability of the cardiovascular system and the cerebral physiology that occurs during laparoscopic surgery.

Our study had several limitations. First, there were limitations in identifying the exact cardiovascular components that distributed the effects of IPC. Changes in vascular compliance or cardiac output were not directly measured but were only estimated based on changes in MAP and HR. Second, since obesity surgery is performed more frequently in female patients, the proportion of female patients in both groups was higher than that of males. Therefore, there is a limit to generalizing the results to all populations, regardless of sex. Since sex may have an effect on cardiac function [13], sex-comparative studies are necessary. Further studies may be needed regarding the utilization of novel devices that may improve hemodynamics and cerebral physiology in obese patients undergoing laparoscopic surgery. The identification of new devices and strategies to help maintain hemodynamic stability in laparoscopic bariatric surgery may encourage the adoption of the laparoscopic approach also in obese patients undergoing emergency general surgery and in the management of postoperative complications after elective bariatric surgery.

## 5. Conclusions

In conclusion, applying IPC to patients with obesity in the steep reverse Trendelenburg position may not improve hemodynamic parameters, vasopressor requirements, or rSO_2_ values during laparoscopic bariatric surgery under lung-protective ventilation. During laparoscopic bariatric surgery, IPC alone has limitations in improving hemodynamics and cerebral physiology. Excess visceral adipose tissue due to obesity increases basal abdominal pressure, reduces venous compliance, and impairs autonomic nervous system activity.

## Figures and Tables

**Table 1:** Patient characteristics and perioperative data.

	Control (*n* = 32)	IPC (*n* = 32)	*p* Value
Age, years	36 ± 8	32 ± 7	0.089
Sex, M/F	6/26	7/25	0.500
Body mass index, kg/m^2^	38 ± 6	38 ± 5	0.825
Anesthesia time, min	183 ± 80	184 ± 81	0.969
Operative time, min	146 ± 74	146 ± 81	0.987
Pneumoperitoneum time, min	118 ± 71	120 ± 81	0.909

Values are presented as the mean ± standard deviation or the number of patients. Control, not applying intermittent pneumatic compression device (IPC); IPC, applying IPC.

## Data Availability

Data are contained within the article.
